# Natural Products as Dietary Agents for the Prevention and Mitigation of Oxidative Damage and Inflammation in the Intestinal Barrier

**DOI:** 10.3390/antiox13010065

**Published:** 2024-01-01

**Authors:** Carlos Martins-Gomes, Fernando M. Nunes, Amélia M. Silva

**Affiliations:** 1 Centre for Research and Technology of Agro-Environmental and Biological Sciences (CITAB), Cell Biology and Biochemistry Laboratory, University of Trás-os-Montes and Alto Douro (UTAD), Quinta de Prados, 5000-801 Vila Real, Portugal; 2 Chemistry Research Centre-Vila Real (CQ-VR), Food and Wine Chemistry Laboratory, University of Trás-os-Montes and Alto Douro (UTAD), Quinta de Prados, 5000-801 Vila Real, Portugal; fnunes@utad.pt; 3 Department of Chemistry, School of Life Sciences and Environment, University of Trás-os-Montes and Alto Douro (UTAD), 5000-801 Vila Real, Portugal; 4 Institute for Innovation, Capacity Building and Sustainability of Agri-food Production (Inov4gro), University of Trás-os-Montes and Alto Douro (UTAD), Quinta de Prados, 5000-801 Vila Real, Portugal; 5 Department of Biology and Environment, School of Life Sciences and Environment, University of Trás-os-Montes and Alto Douro (UTAD), 5000-801 Vila Real, Portugal

**Keywords:** antioxidant, anti-inflammatory, inflammation, intestinal homeostasis, natural products, NF-kB pathway, Nrf2 pathway, oxidative stress, phytochemicals

## Abstract

Food intake is a basic need to sustain life, but foodborne pathogens and food-related xenobiotics are also the main health concerns regarding intestinal barrier homeostasis. With a predominant role in the well-being of the entire human body, intestinal barrier homeostasis is strictly regulated by epithelial and immune cells. These cells are also the main intervenients in oxidative stress and inflammation-related diseases in the intestinal tract, triggered, for example, by genetic/epigenetic factors, food additives, pesticides, drugs, pathogens, and their metabolites. Nevertheless, the human diet can also be seen as a solution for the problem, mainly via the inclusion of functional foods or nutraceuticals that may act as antioxidant/anti-inflammatory agents to prevent and mitigate acute and chronic oxidative damage and inflammation. A literature analysis of recent advances in this topic highlights the significant role of Nrf2 (nuclear factor erythroid 2-related factor 2) and NF-kB (nuclear factor kappa-light-chain-enhancer of activated B cells) pathways in these biological processes, with many natural products and phytochemicals targeting endogenous antioxidant systems and cytokine production and balance. In this review, we summarized and discussed studies using in vitro and in vivo models of the intestinal tract used to reproduce oxidative damage and inflammatory events, as well as the role of natural products as modulators of Nrf2 and NK-kB pathways.

## 1. Introduction 

The essential factors for good health are a healthy lifestyle, physical activity, and a good and healthy diet. A healthy diet is defined as one in which macronutrients are consumed in adequate proportions to meet energetic and physiological needs without excessive intake and which simultaneously provides necessary and sufficient micronutrients and water to meet the body’s physiological needs [1,2]. Food intake is a basic need to sustain life and generally has animal- and/or plant-based sources, providing the macronutrients (carbohydrates, proteins, and lipids) and micro-nutrients (e.g., vitamins and minerals) [1,2]. In addition to the macro- and micronutrients, foodstuff also contain a wide range of other molecules that act as modulators of physiological or pathological processes. Within these molecules, we can not only find hazardous molecules (e.g., pesticides, heavy metals, and toxins) but also molecules that provide health benefits acting in the prevention, mitigation, and/or treatment of several pathologies [3,4,5]. As the gastrointestinal tract is the primary point of contact with foodstuff and respective digestion products, in this review, we emphasize the role of phytochemicals, as nutraceuticals, in the prevention and mitigation of oxidative and inflammatory processes that have been described as factors mediating most of the gastrointestinal diseases.

Being the point of entry of water and nutrients, the gastrointestinal tract is also a hub for a never-ending number of hazardous microorganisms and xenobiotic molecules that accompany foodstuff. Food intake is a basic necessity, a right recognized by the United Nations and supported by the 2030 Agenda for Sustainable Development’s goal to achieve food security, higher nutritional content, and end hunger [6,7]. In developed countries, food access is not a major issue, while food safety and nutrition arise as pending health concerns, as the majority of the population has access to food but presents various setbacks related to intake of a large portion of xenobiotics and possible pathogens. Regarding food safety, among the several sources of contaminations relevant to human health, two arise as relevant in this review: (1) microbial contamination: arising from several microorganisms and their metabolites that cause foodborne illnesses, namely species from *Salmonella*, *Clostridium*, or *Listeria* genus, for example; and (2) chemical contamination: derived from the presence of xenobiotics in food, such as colourants, preservatives, pesticides, or heavy metals [8].

Danger arises from bacteria, enteric viruses, mycotoxins, intentionally or non-intentionally introduced chemicals, and even the existence of food allergies [8,9,10]. Focusing on added chemicals, among food colourants like tartrazine, azorubine, Allura Red, Patent Blue, and others, their safety is partly based on the acceptable daily intake (ADI). Although the intake of these chemicals is low for most of the population and thus does not represent a danger to human health, undesired effects have been reported for some food colourants, and the toxicity of these products is understudied. Also, certain groups within the population may be more exposed than the average population [11]. In addition to this adulteration of foodstuff affecting the final consumer, diet choices also present a huge impact on gut homeostasis. Especially in Western countries, the intake of healthy polyunsaturated fats, antioxidants, and fibre is often substituted by diets rich in cholesterol, saturated fats, and carbohydrates (frequently refined) [12]. Not surprisingly, the high-fat/sugar diet is directly correlated to a higher incidence of non-alcoholic fatty liver disease, while the Mediterranean diet presents the opposite effect and helps in the prevention of chronic diseases [12,13]. The Mediterranean diet offers a variety of food products and is characterized by the consumption of moderate levels of legumes, vegetables, fruits, aromatic plants, whole grains, a variety of nuts and seeds, olive oil, and fish, accompanied by the regular, but controlled, intake of dairy products (e.g., milk, yogurt, kefir, and cheese), and the occasional intake of red and/or processed meats [13], and is considered an equilibrated diet.

The relevance of the factors mentioned above is related to oxidative damage and inflammation in the gut. Considering only internal barriers, the gastrointestinal tract exposure to pathogens and xenobiotic molecules is unmatched [14]. Here, the selective permeability of the intestinal barrier, allowing nutrient and water uptake, but limiting the assess of pathogens and xenobiotics to the systemic circulation, is essential [15]. Its outer layer, the intestinal mucosa, the largest mucosal surface in the human body, presents a contiguous monolayer of columnar epithelial cells. This mucosa is divided into the small and large intestines; the first is still divided into three sections (duodenum, jejunum, and ileum), while the large intestine comprises the cecum, colon, rectum, and anal canal [16,17,18]. The epithelium differs between the two main portions, as the small intestine presents villi and a folded epithelium designed to maximize the area surface and increase nutrient uptake, with crypts (cavities within the folds) and villi (projections of the epithelial cells to increase surface area and specialized in nutrients metabolization and absorption). In the crypts, stem cells can be found, assuring mucosa renewal, proceeding to immature cells and then mature epithelial cells, such as enterocytes, goblet cells, Paneth cells, M (microfold) cells, or enteroendocrine cells [18,19]. It is estimated that the small intestinal epithelium renews every 3–5 days. The large intestine differs in the percentage of each type of cells and lacks the villi [18,19]. Adding to the physical barrier, the intestinal tract also presents a mucus layer (produced by goblet cells), glycocalyx (meshwork formed by the carbohydrate moiety of cell surface glycoproteins), and tight-junction proteins, as well as a chemical barrier with antimicrobial peptides produced by Paneth cells [20,21,22]. This mucosa is kept under a tightly controlled homeostasis and, as a barrier, responds to pathogenic microorganisms and their metabolites, but also to commensal bacteria, food components, and other xenobiotics [23].

The deregulation of the intestinal barrier caused by several exogenous agents, such as pathogens or xenobiotics [15], or by endogenous factors such as genetic predisposition, is a common feature of all intestinal tract pathologies and is frequently described as leaky gut syndrome [24]. In basic terms, when a foreign element (e.g., pathogens, food, or toxins) disrupts the barrier and infiltrates (leaks) to inner layers, it is detected by the immune system and triggers an immune reaction, leading to irritation and inflammation [24]. In pathogen-induced inflammation, the deregulation of tight-junction proteins provides easy access to the inner layers of the mucosa. Cases of food allergies and intolerances are often caused by increased permeability of the intestinal barrier and immune reaction to specific compounds [25]. Adding to the list of potential disruptors of the intestinal barrier are non-steroidal anti-inflammatory drugs (NSAIDs), which are known to induce damage to epithelial cells, disrupt the monolayer, and induce enteropathy, thus leading to the leak of luminal components and triggering of inflammatory cascade and ulceration. This effect is very common since at least 60% of individuals undergoing treatment with NSAIDs present gut deregulation [24,25].

Irritable bowel syndrome is estimated to potentially affect up to 22% of Western countries’ population [15], but it is not the only pathology related to the intestinal barrier, as leaky gut and inflammatory bowel diseases (IBD), which contains ulcerative colitis and Crohn’s disease) also affect a large portion of the population. Risk factors include genetic predisposition, stress, toxins, pesticides, food additives, smoking, unhealthy diet choices, drugs, and others [20,24,26,27]. Oxidative stress is also a contributor and result of IBD and, together with inflammation, is present in the development of another intestinal tract disease: colorectal cancer. In fact, individuals with IBD are more likely to develop colorectal cancer, like in the case of ulcerative colitis-associated cancer [28,29,30]. Colorectal cancer occurrence is higher in Western countries and is the third deadliest cancer, with the incidence rate growing each year. Most of the cases are sporadic, but genetic factors also contribute to the diagnosed cases [29,31]. Oxidative stress on its own presents a significant risk to the development of colorectal cancer. The unbalance of pro-oxidant species vs. cells’ antioxidant potential, favouring the rise in pro-oxidant molecules, triggers oxidative stress, and is characterized by an increase in reactive oxygen (ROS) and nitrogen (RNS) species, such as superoxide radical (O_2_^•−^), hydroxyl radical (^•^OH), hydrogen peroxide (H_2_O_2_), peroxyl (ROO^•^), nitric oxide (NO), or peroxynitrite (ONOO^−^). These interact with proteins, nucleic acids, and lipids, leading to mutagenesis, cell damage and, relevant to this topic, colorectal cancer onset [30]. 

**Figure 1:**
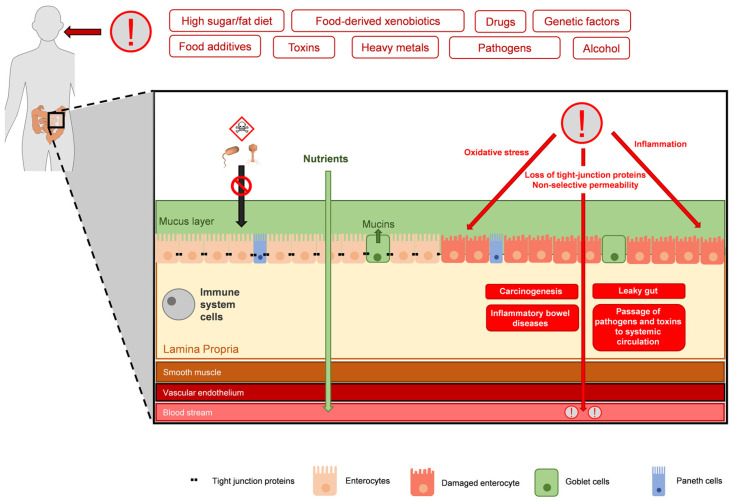
Schematization of the intestinal mucosa anatomy; major functions (nutrient uptake and defence against pathogens/xenobiotics) and main endogenous and exogenous risk factors contributing to common pathologies in the intestinal barrier.

Most important is the notion that some dietary components (e.g., fruits, vegetables, and aromatic and medicinal plants) present antioxidant and anti-inflammatory activities and are known to reverse leaky gut syndrome and restore barrier homeostasis [24,32]. The intestinal tract is the potential point of entry for hazardous elements that compromise the barrier function and subsequently affect internal organs, but its main function is nutrient uptake, and many sources of nutrients are also sources of phytochemicals. The proposed strategy is to add antioxidant and anti-inflammatory natural products to the diet as functional foods and nutraceuticals, aiming for the maintenance of intestinal mucosa homeostasis and the prevention of oxidative damage, inflammation, and related pathologies [33]. In this review, we summarize and discuss the potential impact of certain natural products as dietary agents with the potential to regulate intestinal homeostasis and prevent oxidative stress- and inflammation-related intestinal pathologies. Figure 1 summarizes the main risk factors for increased oxidative stress and inflammation in the intestinal tract.

## 2. Antioxidant and Anti-Inflammatory Activity of Natural Compounds at Intestinal Level

Food is considered one of the three foundational pillars of human beings’ health and survival (the others being socio-mental engagement and physical exercise), which, in a holistic view, appears to successfully contribute to maintaining health and extending longevity [34]. An unhealthy lifestyle is known to exacerbate oxidative stress, where diet, alcohol, and smoking play a significant role, but it is also affected by exposure to toxins, by the existence of metabolic diseases, or by dysbiosis [35]. The persistent oxidative state is then correlated to chronic inflammation and oncological diseases onset [36]. However, the quality and quantity of consumed food influence optimal nutrition for human health and survival. In the next subsections, the relevance of food components (hazardous and beneficial ones) concerning the balance between oxidative stress and antioxidant action, as well as the balance between inflammation and anti-inflammatory action, will be presented and discussed. Also, the effect of natural products (extracts or individual compounds) on improving or mitigating oxidative or inflammatory processes caused by different agents via the modulation of specific cellular pathways will also be addressed and discussed.

### 2.1. Antioxidant Potential of Extracts against Xenobiotics and Contaminants Resulting from Food Processing

Individual food intake and preferred types of diet play a significant role in the exposure to an ever-increasing number of xenobiotics introduced into the human diet, mainly due to excessive consumption of processed foods, potentially toxic molecules originating during food processing or synthetic additives that are often used as sweeteners or preservatives. An example of xenobiotic resulting from foodstuff processing is acrylamide, a product of the Maillard reaction that occurs in carbohydrate-rich foods when cooked at high temperatures. Bread, breakfast cereals, coffee products, french fries, and potato chips are known to contain acrylamide, which is listed as carcinogenic [37,38,39]. Particularly at the intestinal level, acrylamide was shown to accelerate the development of ulcerative colitis in mice, presenting reduced levels of glutathione (GSH), catalase (CAT), superoxide dismutase (SOD), and IL (interleukin)-10 [40]. In addition, increased lipid peroxidation, carbonyl protein groups, and nitric oxide (NO), as well as tumour necrosis factor-alpha (TNF)-α, IL-6, IL-1β, interferon-gamma (IFN)-γ, nuclear factor kappa-light-chain-enhancer of activated B cells (NF-kB), and inducible nitric oxide synthase (iNOS) expression was observed in mice pre-exposed to acrylamide prior to colitis induction, thus displaying several increased biomarkers of oxidative stress and inflammation [40]. A different study confirmed these results using a cell line model of human colon epithelium (Caco-2 cells), where acrylamide induced an increase in ROS and pro-inflammatory cytokines (TNF-α, IL-6, and IL-1β) and a decrease in the anti-inflammatory cytokine IL-10 [41]. Furthermore, acrylamide reduced the expression of claudin-1, occludin, and ZO-1 (zonula occludens 1), which contribute to higher permeability through the intestinal barrier [41]. In addition to acrylamide, the authors reported similar results to ochratoxin A, a toxin and food contaminant produced by some species of *Aspergillus* and *Penicillium* genus, frequently found in cereals (e.g., maize, wheat, and barley) and derivatives such as flour or coffee [41]. Phytochemicals have been proposed as effective countering agents of acrylamide toxicity, namely countering oxidative stress and inflammation in various tissues [39]. Specifically in the intestinal tract, crocin (a carotenoid identified in *Crocus Sativus* L. flowers, from which saffron is produced) was shown to decrease lipid peroxidation and increase total antioxidant capacity and GSH levels in small and large intestine samples of rats exposed to acrylamide [37]. Additionally, crocin normalized SOD and CAT levels while also preventing acrylamide-induced degeneration of the villi [37]. Also presenting the capacity to mitigate acrylamide-induced oxidative stress, both a cocoa extract and two of its main components, epicatechin and procyanidin B2, were shown to reduce oxidative stress induced by acrylamide via the prevention of GSH depletion and upregulation of GST (glutathione *S*-transferase) and GCL (glutamate-cysteine ligase; also known as GCS: γ-glutamylcysteine synthetase), preventing cell death, as observed by reduced caspase-3 activation and increased cell viability [42]. The antioxidant effect of procyanidin B2 was higher than epicatechin, and the cocoa extract presented higher antioxidant potential than the individual components [42], which may indicate a synergistic effect. In acrylamide-induced inflammation, a protective effect was observed for allicin, an organosulphur component of garlic [43]. Analyzing colon samples from Sprague Dawley rats exposed to acrylamide and allicin (25 or 50 mg/kg/day), it was reported that allicin increased the expression of tight-junction proteins (claudin-1, occludin, and ZO-1), as well as mucin-2 and mucin-3 [43]. The levels of LPS (lipopolysaccharides) and the inflammatory cytokines IL-1β, IL-18, TNF-α, and IL-6 were also decreased by allicin in addition to IL-10 increase [43].

Among the toxic components found in foodstuff, oxysterols are described as pro-oxidant and carcinogenic [44,45,46]. These products of cholesterol oxidation, such as 7α-hydroxycholesterol or 7β-hydroxycholesterol, are found mainly in eggs and milk-based products and are known to induce oxidative stress and inflammation in the intestinal tract [44,45,46]. Of interest for the subject of this review is the ability of certain natural products in preventing the pro-oxidant and pro-inflammatory activity of oxysterols, which is the case of the phenolic compounds present in extra virgin olive oil [45,46]. This food product, highly present in the Mediterranean diet, presents a high content in hydroxytyrosol, tyrosol, 3,4-dixydroxyphenylethanol elenolic acid (3,4-DHPEA-EA), 2-(4-hydroxyphenyl)ethyl (*E*)-4-formyl-3-(2-oxoethyl)hex-4-enoate (*p*-HPEA-EDA), methyl (4*S*,5*Z*,6*R*)-4-[2-[2-(3,4-dihydroxyphenyl)ethoxy]-2-oxoethyl]-5-ethylidene-6-hydroxy-4*H*-pyran-3-carboxylate (3,4-HPEA-EA), or 2-(4-hydroxyphenyl)ethyl-(*E*)-4-formyl-3-(2-oxoethyl)hex-4-enoate (*p*-HPEA-EA), for example, and was shown to reduce the level of ROS and prevent the decrease in GSH induced by exposure to *tert*-butyl hydroperoxide or by an oxysterols mixture (7-ketocholesterol, 7α-hydroxycholesterol, 7β-hydroxycholestrol, 5α,6α-epoxycholesterol, and 5β,6β-hydroxycholesterol) [45,46]. In addition, lipid peroxidation and content in fatty acid hydroperoxides were also decreased by the phenolics present in olive oil samples [45,46]. Also relevant was the effect of these phenolic compounds on intestinal inflammation, as the phytochemicals were able to reduce oxysterols-induced increase in NO (nitric oxide), IL-6, and IL-8, as well as reduce JNK (c-Jun *N*-terminal kinases), IkB (IkappaB kinase) and p38 (p38 mitogen-activated protein kinases) phosphorylation, and iNOS expression [46].

These beneficial effects of natural compounds were also observed to mitigate herbicide-induced damage. For example, an essential oil obtained from *Origanum vulgare* L. was assessed for its potential to prevent oxidative damage induced by the herbicide diquat (IUPAC name: 7,10-diazoniatricyclo[8.4.0.02,7]tetradeca-1(14),2,4,6,10,12-hexaene) in Wistar rats’ intestinal tract [47]. Diquat, often used as diquat dibromide, is known to produce superoxide anion radicals and hydrogen peroxide, which are most likely on the basis of its oxidative damage [47]. The oregano essential oil tested was effective in reducing diquat-induced ROS and lipid peroxidation in jejunum samples, namely via the regulation of the endogenous antioxidant enzymes SOD and GPx, whilst also improving the barrier integrity via the increased expression of tight-junction proteins occludin and ZO-1 [47]. The potential of *O. vulgare* essential oils as antioxidant agents in the intestinal tract was also addressed by Zou et al., 2016 [48], who reported the protective effect against H_2_O_2_-induced oxidative damage in porcine small intestinal epithelial cells (IPEC-J2). At 10 µg/mL, the essential oil reduced both intra- and extracellular ROS, reduced lipid peroxidation (observed as malondialdehyde (MDA) reduction assay), and increased intracellular GSH content [48]. To explore the mechanisms of action behind this bioactivity, the authors analyzed the mRNA and protein content of SOD, CAT, GCL, and Nrf2 (nuclear factor erythroid 2-related factor 2), which were upregulated by the essential oil. As a major transcription factor involved in response to oxidative damage, Nrf2 activation was further studied, and it was observed that the essential oil induced Nfr2 translocation to the nuclei. In addition, the previous increase in SOD and GCL expression and intracellular GSH was reduced when Nfr2 was intentionally downregulated, thus supporting the key role of Nfr2 in the antioxidant response [48].

### 2.2. Antioxidant Potential of Extracts against High Caloric Diet

In addition to xenobiotics, the overconsumption of sugars and fats also presents a significant health concern. Fructose, for example, is used in the food industry as a sweetener in many processed products, and a high intake of this monosaccharide is known to contribute to various liver pathologies, such as non-alcoholic fatty liver disease, but also to obesity and diabetes [49]. Concerning oxidative stress, a high fructose diet in C57BL/6J mice was reported to increase lipid peroxidation and reduce GSH levels and SOD activity in hepatic tissue [49]. In addition, a high fructose diet reduced the levels of phosphorylated Akt (p-Akt) and Nrf2 and increased the levels of p-JNK. Concerning hepatic inflammation, fructose increased the expression of NF-kB, TRIF (TIR-domain-containing adapter-inducing interferon-β), TLR4 (Toll-like receptor 4), MyD88 (myeloid differentiation factor 88), and serum TNF-α [49]. At the intestinal level, it was observed that the increased content of fructose reduced the expression of tight-junction proteins, mucin-2 and -4, and increased the expression of TLR4, TLR5, and NF-kB [49]. With particular interest, the same study reported the beneficial effect of loquat (*Eriobotrya japonica* (Thunb.) Lindl.) fruit hydroethanolic (20:80; *v*/*v*) extract, rich in chlorogenic, cryptochlorogenic and oleanolic acids, and in phloretin and hesperidin. This extract (at 25 and 50 mg/kg) was efficient in preventing the increase in body weight and fat induced by excessive fructose intake and, at the hepatic level, it normalized Akt expression and phosphorylation, reduced JNK phosphorylation and lipid peroxidation, and increased SOD activity and GSH content [49]. Regarding inflammation markers, serum TNF-α levels were reduced by loquat fruit extract, as well as liver levels of TLR4-associated proteins and NF-kB. These were later also observed at the intestinal level, in addition to increased expression of tight-junction proteins and mucins, when compared to mice only treated with a high-fructose diet [49]. Also exploring the role of phytochemicals as a tool to prevent diet-induced oxidative stress in the intestinal tract, Fernando et al., 2015 [50] studied the protective role of a standardized cranberry extract (acquired from Nutra Canada, Champlain, QC, Canada), rich in flavonols and proanthocyanidins, in reducing oxidative damage induced by a high fat/high sucrose diet. At the intestinal level, the extract reduced triglyceride accumulation and increased SOD2 levels while having no effect on GPx (glutathione peroxidase) and malondialdehyde levels [50]. Nevertheless, at the hepatic level, in addition to an increase in SOD2, GPx activity was also increased, and a reduction in lipid peroxidation was observed [50].

Regarding the control of obesity, using as model Sprague Dawley rats on a high-fat diet, a study reported the potential of Fu brick tea (a product of microbial fermentation of *Camellia sinensis* L. leaves, the material traditionally used to produce tea), rich in epigallocatechin gallate, epigallocatechin, and epicatechin [51]. Fu brick tea was effective in reducing the oxidative stress induced by a high-fat diet in the colon, namely via SOD- and CAT-increased activity [51]. The extract’s ability to regulate intestinal epithelium homeostasis was also supported by the upregulation of ZO-1, claudin-1, and occludin, together with morphologic analysis of duodenum, jejunum, ileum, and colon histological sections, where a control-like morphology was observed [51].

### 2.3. Antioxidant Potential of Extracts against Commonly Used Food Additives

Food additives, for example, present yet another source of stress to the intestinal barrier. Dorier et al., 2017 [52] reported moderate toxicity induced by titanium dioxide (TiO_2_), a food additive also addressed as E171, used as a whitening and opacifying agent [52]. The authors report increased ROS in both the Caco-2 monolayer and Caco-2/HT-29MTX (human colorectal adenocarcinoma cells that differentiate into mucus-secreting cells induced by methotrexate) co-culture exposed to TiO_2_, in both the short exposure times (6 h, 24 h, or 48 h) and in repeated exposure for 21 days. Related to the increase in reactive species is the reduced gene expression of genes encoding CAT, GR, SOD1, SOD2, and Nfr2. The oxidative damage was notorious via DNA damage [52].

Noteworthy, despite Caco-2 cells being derived from a human colorectal adenocarcinoma, this cell line is widely used in studies related to intestinal barrier damage since, unlike other colorectal cell lines, Caco-2 cells do not present mutation in genes related to PI3K/Akt/mTOR (PI3K: phosphoinositide 3-kinases; Akt: protein kinase B; and mTOR: mammalian target of rapamycin) or in the RAS–RAF–MAPK (RAS: from “rat sarcoma virus”, GTPase; RAF: from “rapidly accelerated fibrosarcoma”, serine/threonine-specific protein kinases; and MAPK: mitogen-activated protein kinase) signalling axis [53]. In addition, Caco-2 cells present enterocyte-like differentiation, typically starting after 7 days of culture and taking up to 21 days, which simulates intestinal epithelium, presenting microvilli formation, typical thigh-junction proteins expression, brush border structure, and expression of enzyme and transporter systems that allow permeability, antioxidant, and anti-inflammatory studies [53,54], particularly with foodstuff, since the intestinal tract is the first barrier capable of mediating nutrient uptake and defence against xenobiotics.

Table 1 summarizes natural compounds that present the potential to mitigate oxidative stress in the intestinal tract, evaluated in various in vitro and in vivo experimental models, and induced by a variety of oxidative agents.

The toxicity of food additives to the intestinal barrier is displayed in many forms. It is reported that artificial sweeteners such as aspartame deregulate intestinal permeability, downregulate tight-junction proteins, and increase ROS in Caco-2 cells [60]. Another artificial sweetener, saccharin, was shown to disrupt Caco-2 cells monolayer integrity with the involvement of NF-kB upregulation and reduced claudin-1 expression [61]. Acesulfame potassium also increased intestinal permeability and increased the expression of IL-1β, TNF-α, and IFN-γ in C57BL/6J mice small intestine while also increasing lymphocyte migration to the mucosa [62]. Dietary emulsifiers are also studied for their toxicity in the intestinal tract [63]. Both P-80 (polysorbate 80) and maltodextrin were shown to promote intestinal inflammation [64,65]. Food preservatives are also on this list. An example is sodium bisulphite, which increased ROS levels and lipid peroxidation, and reduced SOD and GSH levels in a normal human colon mucosal epithelial cell line (NCM460) [66]. Regarding inflammation, sodium bisulphite induced the expression of NF-kB and induced the expression of IL-6 and TNF-α [66]. However, this is not a solitary example, as various other food preservatives have been reported to induce an increase in inflammatory cytokines, as is the example of butylated hydroxyanisole (BHA), sodium benzoate, or boric acid [67]. Given the daily exposure of the general population to food additives, the necessity to find dietary agents that can mitigate the oxidative and inflammatory damage of these xenobiotics is mandatory.

### 2.4. Antioxidant Potential of Extracts against Other Diet or Ingested Components

Alcohol ingestion is also a major health concern. In the intestinal barrier, alcohol is shown to induce oxidative stress and increased permeability, as well as inflammation, with increased expression of inflammatory cytokines [68,69]. Once again, dietary natural products present themselves as therapeutic agents to reverse the alcohol effect. For example, garlic oil was able to reduce alcohol-induced oxidative stress and lipid peroxidation in Sprague Dawley rats by increasing SOD and GPx levels in the colon and to upregulate ZO-1 and claudin-1 expression [57]. Orange peel extract was also evaluated for its protective activity over ethanol exposure using the Caco-2 cells monolayer model. The extract prevented an increase in intestinal permeability caused by alcohol, with the upregulation of tight-junction proteins [70]. In alcohol-induced colon inflammation, a polyphenol-rich extract of Zhenjiang aromatic vinegar was shown to increase IL-10 and IL-22 levels in the colon of ICR (Institute of Cancer Research) mice exposed to ethanol and also to reduce TNF-α, IL-6, IL-1β, and LPS levels [71].

Despite the scarcity of studies reporting the potential of natural compounds to prevent or mitigate the oxidative and inflammatory damage caused by the hazardous agents mentioned above, many studies have explored the antioxidant potential in response to standard oxidative agents, such as peroxides, as shown in Table 1 and Table 2. For example, resin/gum extracted from *Boswellia serrata* was reported to protect Caco-2 cells against H_2_O_2_-induced oxidative damage [59]. It was observed that ROS levels were decreased, and the upregulation of NF-kB induced by H_2_O_2_ was reversed. The protective effect of this natural product was also supported by the analysis of ZO-1 and occludin, which demonstrated that exposure to *B. serrata* resin extract was effective in maintaining tight-junction proteins expression and contributed to a normal Caco-2 monolayer morphology [59]. In this same study, Catanzaro et al., 2015 [59] also reported an identical effect promoted by one of *B. serrata* resin components, 3-*O*-acetyl-11-keto-β-boswellic acid, which also presented antioxidant potential in Caco-2 cells at a very low concentration (27 ng/mL), thus being possible to correlate the observed bioactivity to the phytochemical composition. Due to the effect of both the resin and its component on tight-junction proteins, the paracellular permeability induced by oxidative damage also presented significant reduction [59].

### 2.5. Antioxidant Potential of Phytochemicals Commonly Found in the Diet

A pattern often observed in studies using phytochemicals is related to their antioxidant vs. prooxidant behaviour. Despite being reported as antioxidants, an attribute mainly validated in synthetic radicals’ scavenging assays, in biological systems, the antioxidant potential is less likely to increase proportionally with the concentration, as at higher concentrations, the phytochemicals start to induce toxicity, often triggered by ROS increase, thus supporting the general conception that “the dose makes the poison”.

An example is provided by Llana-Ruiz-Cabello et al., 2015 [72], using carvacrol, a terpene usually found in essential oils obtained from some plants belonging to the Lamiaceae family. Firstly, the authors report that Caco-2 cells exposed to concentrations above 230 µM present increased ROS levels and GSH depletion. However, at concentrations ranging between 53 and 214 µM, carvacrol was able to protect against oxidative stress induced by 100 µM H_2_O_2_ and also prevented GSH depletion [72]. This highlights the need to assess the safety profile of natural products and their components, as bioaccumulation may increase in cases of extended exposure and thus accumulate at levels above the therapeutic concentrations, presenting potential toxicity.

Various individual phytochemicals have been assessed for their antioxidant potential. Epigallocatechin gallate (EGCG) was able to reduce ionizing radiation-induced oxidative damage in the intestinal tract of C57BL/6J mice [73]. Upon EGCG treatment, the small intestine epithelium was able to retain its normal morphology (evaluated as villi’s height and crypt’s depth) and reduced DNA damage was observed [73]. In HIEC cells (human intestinal epithelial cells), EGCG enhanced the expression of Nrf2 to mitigate ionizing radiation-induced ROS, with increased expression of downstream targets of Nrf2, such as HO-1 [73].

Resveratrol, a stilbenoid commonly found in grapes, various berries and peanuts [74], was shown to significantly ameliorate H_2_O_2_-induced oxidative damage in a porcine intestinal cell model, being proposed as a potential additive in livestock feed to avoid intestinal damage [75]. As reported, while presenting low/no toxicity to IPECJ2 cells at concentrations up to 50 µM, resveratrol reduced H_2_O_2_-induced apoptosis and necrosis, as confirmed by annexin V-FITC/PI double staining assay [75]. A decrease in ROS content was observed, accompanied by increased activity of CAT, GPx, and SOD, upregulation of Keap1 (Kelch-like ECH-associated protein 1), and increased phosphorylation of Nrf2 and Akt proteins. In addition, the effect of resveratrol was also extended to tight junction proteins claudin-1, occludin, and ZO-1 (tight junction protein-1), essential to maintain intestinal barrier function [75]. Curcumin was able to reduce H_2_O_2_-induced oxidative damage in IPECJ2 cells via the upregulation of SOD, CAT, and GPx [76]. The expression of claudin-1 and ZO-1 was increased, revealing the potential of curcumin to mitigate oxidative damage in the intestinal tract [76]. The antioxidant effect was via the induction of Parkin-dependent mitophagy and of the adenosine monophosphate-activated protein kinase (AMPK)/transcription factor EB (TFEB) pathway [76].

Similar to the above described for resveratrol potential as a supplement for animal feed [75], similar findings were reported for a mixture of two terpenoids, carvacrol and thymol [77]. Using weaning piglets (piglets to which adult diet was introduced replacing breast milk) as a model to trigger stress in the intestinal tract, it was shown that supplementing the diet with carvacrol–thymol reduced oxidative stress induced by the weaning process as observed by decreased ROS and lipid peroxidation, as well as increased activity of endogenous antioxidant enzymes SOD and GPx [77]. In addition, the supplementation contributed to the intestinal barrier integrity, observed as increased occludin expression, and also to an anti-inflammatory status as shown by the decrease in TNF-α and IL-1β [77].

Table 2 presents a summary of phytochemicals evaluated for their antioxidant potential in in vitro and in vivo experimental models.

**Table 2:** Antioxidant activity of various phytochemicals evaluated using in vitro and in vivo experimental models of the intestinal tract.

Compound	Concentration	Experimental Model	Observations	Ref.
Caffeic acid	250 mg/kg	Intestinal samples from Wistar rat	Decreased cisplatin-induced lipid peroxidation Increased SOD, GST, GR, GPx, and CAT activities	[78]
Crocin	50 mg/kg	Ileum and colon samples from Wistar Rats	Reduced acrylamide-induced oxidative stress Reduced lipid peroxidation Normalized SOD and CAT levels Increased GSH levels Prevented villi degradation	[37]
Caffeic acid	60 and 120 mg/kg	Intestinal sample from Sprague Dawley rats	Reduced ketoprofen-induced oxidative damage Increased GPx and GR activities Increased GSH content HO-1 upregulation	[79]
Carvacrol-thymol mixture	100 mg/kg (1:1)	Jejunum samples from swine	Decreased weaning-induced intestinal oxidative stress Decreased ROS levels and lipid peroxidation Increased SOD and GPx activity	[77]
Ellagic acid	10 mg/kg	Jejunum samples from BALB/c mice	Reduced oxidative stress induced by oxidized fish oil Reduced lipid peroxidation Increased SOD and GPx activity	[80]
Punicalin				
Punicalagin				
Puerarin	10 and 50 mg/kg	Colon samples from BALB/c mice	Reduced dextran sulphate sodium-induced oxidative stress Reduced lipid peroxidation Prevented GSH depletion Normalized SOD and CAT activity Normalized Nfr2, HO-1, and NQO1 expression	[81]
Eriodictyol	20 and 50 mg/kg	Colon samples from Wistar rats	Prevented 2,4,6-trinitrobenzenesulfonic acid-induced reduction in SOD, CAT, and GPx levels Increased IL-10 levels Reduced lipid peroxidation	[82]
Epigallocatechin gallate	50 mg/kg	Colon samples from C57BL/6J mice	Reduced dextran sulphate sodium-induced oxidative damage Reduced lipid peroxidation Increased SOD and GPx levels	[83]
	25 mg/kg	Small intestine samples from C57BL/6J mice	Prevented morphological alterations induced by ionizing radiation	[73]
	2 µM	Human intestinal epithelial cells (HIEC)	Reduced ROS induced by ionizing radiation Upregulated Nrf2 and HO-1	[73]
Chlorogenic acid	25 µM	Porcine intestinal epithelial cells (IPEC-J2)	Reduced extracellular H_2_O_2_ content and intracellular ROS levels induced by LPS	[84]
3-Acetyl-11-keto-β-boswellic acid	27 ng/mL	Human colorectal adenocarcinoma cells (Caco-2)	Reduced H_2_O_2_-induced ROS increase and NF-kB expression Prevented downregulation of tight-junction proteins (ZO-1 and occludin)	[59]
Resveratrol	50 µM	Porcine intestinal epithelial cells (IPECJ2 cells)	Reduced H_2_O_2_-induced cell death Reduced oxidative stress Increased CAT, GPx, and SOD expression and activities Reversion of H_2_O_2_-induced downregulation of claudin-1, occludin and ZO-1 Upregulation of Nrf2, Akt, and Keap1	[75]
Curcumin	50 µM	Porcine intestinal epithelial cells (IPECJ2 cells)	Reduced H_2_O_2_-induced cell death Decreased ROS and lipid peroxidation Increased SOD and CAT levels Increased SOD and GPx expression	[76]
Carvacrol	53.5–214 µM	Human colorectal adenocarcinoma cells (Caco-2)	Decreased H_2_O_2_-induced oxidative stress Avoided GSH depletion	[72]
Thymol	62.5–250 µM			
Procyanidin B2	10 µM	Human colorectal adenocarcinoma cells (Caco-2)	Reduced acrylamide-induced oxidative stress and cell death Prevented GSH depletion Increased GST and GCL levels	[42]
Epicatechin	10 µM	Human colorectal adenocarcinoma cells (Caco-2)	Reduced acrylamide-induced oxidative stress and cell death Prevented GSH depletion and decreased GST and GCL levels	[42]
Caffeic acid	50 µM	Human intestinal epithelial cells (Int-407)	Reduced ketoprofen-induced ROS Increased GPx and GR activities Nrf2, DJ-1, and HO-1 upregulation	[79]
Schisandrin A	10 µM	Human colorectal adenocarcinoma cells (HT-29)	Reduced deoxynivalenol-induced oxidative stress Increased CAT, SOD, GPx, and GST activity Increased GSH content	[85]

Abbreviations (not defined previously): DJ-1, human protein deglycase (encoded by *DJ-1* gene, also known as *PARK7*).

An additional protective effect with pharmaceutical applications is the ability to protect against the oxidative stress induced by drugs of oral administration, as is the case of various NSAIDs [86]. Caffeic acid, for example, is able to preventively protect the intestinal tract of Sprague Dawley rats against the oxidative damage caused by ketoprofen, an effect also observed in an in vitro model of human intestinal epithelial cells (Int-407) [79]. In both models, 50 µM of caffeic acid reduced ROS and increased GPx and GR levels [79]. In Int-407 cells, the effect was mediated by the activation of the Nrf2 pathway and increased expression of protein deglycase DJ-1, leading to increased expression of HO-1 [79]. The expression of HO-1 was also increased in the intestinal mucosa of the rat model [79]. Additionally, caffeic acid also reduced COX-2 expression and nitric oxide production in the in vivo model [79]. Among the various classes of phytochemicals with pharmacological value, flavonoids must also be highlighted for their antioxidant potential evaluated in animal experimental models. An example is puerarin, a derivative of daidzen that presents a *C*-linked glucoside in position 8, found in the roots of *Pueraria lobata* (commonly known as kudzu) [81]. In a model of colitis induced by dextran sulphate sodium (DSS) in BALB/c mice, puerarin was able to reduce oxidative stress in the colon, with emphasis on reduced lipid peroxidation and the regulation of endogenous enzymatic systems (SOD and CAT activity normalization; prevention of GSH depletion), which were observed to be dependent on normalization of Nfr2, HO-1, and NQO1 expression [81]. In addition, intestinal barrier function was preserved in the presence of puerarin, supported by the upregulation of tight-junction proteins ZO-1, occludin, and claudin-1, reported by both protein and mRNA expression increases [81]. As will be addressed below, puerarin also presented anti-inflammatory potential in the same model.

### 2.6. Anti-Inflammatory Potential of Plant-Derived Extracts

Anti-inflammatory activity is also highly addressed for various natural products and their phytochemicals, as seen above. Table 3 summarizes studies reporting the anti-inflammatory effect of several natural products, administered as extracts, in in vitro and in vivo models of intestinal inflammation.

Romier-Crouzet et al., 2009 [87] evaluated the anti-inflammatory potential at the intestinal level of diverse natural products, namely pomegranate, cocoa, sugar cane, grape seeds, and oak (duramen) extracts. Aiming to normalize the bioactivities observed by their polyphenolic contents, the authors initially quantified the total phenolic content of each extract and then proceeded to cell-based assays using the concentration of 50 µM gallic acid equivalents (GAE). Nevertheless, it is worth mentioning that the extract that showed the highest potential to modulate several inflammatory pathways (pomegranate; *Punica granatum* L. fruit peel aqueous extract) is also the extract that presents the lowest total phenolic content (108.2 mg GAE/g DW), thus making the ingestion of a much higher quantity necessary when compared to other products tested that also present significant anti-inflammatory activity, namely grape seed extracts (640.5 mg GAE/g DW) or sugar cane extract (276.2 mg GAE/g DW) [87]. Of all products tested, *Garcinia mangostana* fruit peel aqueous extract was the only one that did not present any anti-inflammatory potential, as evaluated in Caco-2 cells stimulated with IL-1β or with a cytokine cocktail (IL-1β, TNF-α, and IFN-γ) plus LPS. On the other hand, pomegranate peel extract presented the potential to modulate the largest number of targets within the inflammatory cascade, namely reducing ERK 1/2 activation (extracellular signal-regulated kinase), NO release, and IL-1β-induced NF-kB activation and IL-8 and PGE_2_ (prostaglandin E2) secretion [87]. None of the other extracts was able to reduce ERK 1/2 activation. However, *Saccharum officinarum* L. stem (sugar cane) and *Quercus robur* L. (oak) duramen extracts also present significant anti-inflammatory activity, selectively targeting the signalling pathways mentioned above [87]. Of interest were the results obtained for PGE_2_ secretion inhibition by *Theobroma cacao* L. (cocoa plant) extract, showing that in Caco-2 cells non-stimulated with IL-1β, the extracts induced PGE_2_ secretion (3.7-fold increase), whilst in IL-1β-stimulated cells, a reduction in PGE_2_ secretion (2.2-fold decrease) was observed [87]. The authors hypothesize that this is due to COX-1 (cyclooxygenase 1) activation in cells not exposed to cytokines, as COX-1 is involved in intestinal barrier integrity regulation, and is essential in its homeostasis. In the presence of cytokines, COX-2 (cyclooxygenase 2), an inducible key enzyme in the inflammatory cascade, is responsible for the increased production of PGE_2_ observed in the positive control, but in this case, it is inhibited by *Theobroma cacao* extract [87]. This highlights the need to further study the selective inhibition/activation of COX isoforms for intestinal barrier regulation and inflammation inhibition.

As mentioned above in Table 1 for its antioxidant potential observed in a sodium sulphate dextran-induced colitis model, the grape seed proanthocyanidin extract studied by Sheng et al., 2020 [56] also presented significant anti-inflammatory potential (Table 3). The extract reduced the levels and the mRNA expression of the inflammatory cytokines TNF-α and IL-1β, whilst increasing the anti-inflammatory cytokine IL-10. The authors also inferred that the extract might be targeting the inflammasome formation (a significant target under study for the treatment of colitis) as a reduction in the key proteins NLRP3 (NOD-, LRR-, and pyrin domain-containing protein 3; NOD, nucleotide-binding oligomerization domain; LRR, leucine-rich repeat), ASC (apoptosis-associated speck-like protein containing a CARD), and caspase-1 was observed. In addition, the extract increased the expression of tight junction proteins (ZO-1, occludin, and claudin), contributing to intestinal epithelium resistance to the oxidative and inflammatory stimulus [56].

In the section above addressing intestinal oxidative stress, the role of high-sugar and high-fat diet on oxidative damage was mentioned, as well as reports on natural products with the potential to prevent it. The same potential was also observed for inflammation prevention with a standardized cranberry extract, which was able to reduce serum LPS, while also decreasing COX-2 and NF-kB in the jejunum of male C57BL/6J mice maintained in a high-fat/high sucrose diet, but presenting no effect on TNF-α levels [50]. Fu brick tea, above mentioned for the antioxidant potential (Table 1), also presents potential as a dietary anti-inflammatory agent, in effect mediated by increased levels of the anti-inflammatory cytokine IL-10 and reduced expression of pro-inflammatory cytokines IL-6 and TNF-α, as well as reduced expression of MCP-1 (monocyte chemoattractant protein 1; also commonly referred as chemokine (C-C motif) ligand 2 (CCL2)), a chemokine responsible for the recruitment of immune cells to the inflammation site [51]. Remarkably, the extract also reduced LPS, inflammatory cytokines, and chemokines in the serum, in addition to its potential as a prebiotic agent to treat obesity via gut microbiota modulation [51].

### 2.7. Anti-Inflammatory Potential of Phytochemicals Commonly Found in the Diet

The anti-inflammatory activity of individual compounds is directly correlated with the anti-inflammatory potential of the complex matrices, such as the extracts presented in Table 2. This effect was observed for various phenolic acids, terpenes, flavonoids, and other classes of phytochemicals. Table 4 summarizes studies reporting the anti-inflammatory effect of several phytochemicals in in vitro and in vivo models of intestinal inflammation.

As mentioned above in Table 2, for its antioxidant activity in the colon of Balb/c mice with dextran sulphate sodium-induced colitis, the isoflavonoid puerarin also presented anti-inflammatory activity, evaluated in the same samples [81]. A comprehensive study of various biomarkers of inflammation reports a reduction of NO and PGE_2_ in mice treated with puerarin, which was correlated to the reduction in iNOS and COX-2 protein expression and mRNA levels, respectively. In addition, puerarin also decreased the mRNA levels of several cytokines, namely TNF-α, IFN-γ, IL-1β, and IL-6 [81].

**Table 4:** Anti-inflammatory potential of natural products, administered as individual compounds, at intestinal level.

Phytochemical	Concentration	Experimental Model	Observations	Ref.
Carvacrol-thymol mixture	100 mg/kg (1:1)	Jejunum samples from swine	Decreased TNF-α and IL-1β mRNA levels in weaning piglets	[77]
Ellagic acid	10 mg/kg	Jejunum samples from BALB/c mice	Decreased TNF-α, IFN-γ, and IL-6 mRNA expression induced by oxidized fish oil	[80]
Punicalin			Decreased TNF-α, IFN-γ, and IL-6 mRNA expression induced by oxidized fish oil	
Punicalagin			Decreased IFN-γ mRNA expression induced by oxidized fish oil	
Puerarin	10 and 50 mg/kg	Colon samples from BALB/c mice	Reduced DSS-induced TNF-α, IFN-γ, IL-1β, and IL-6 mRNA expression Reduced NO and PGE_2_ production Reduced COX-2 and iNOS protein and mRNA expression	[81]
Curcumin	100 mg/kg	Colon samples from BALB/c mice	Reduced DSS-induced inflammation Reduced iNOS expression and NO production Decreased TNF-α, IL-1β, and IL-6 mRNA expression Reduced NF-kB activation	[88]
	100 mg/kg	Colon samples from Sprague Dawley Rats	Reduce 2,4,6-trinitrobenzenesulfonic acid-induced colitis Reduced expression of NF-kB and IL-27 mRNA Decreased protein expression of TLR4, NF-kB, and IL-27	[89]
Epigallocatechin gallate	50 mg/kg	Colon samples from C57BL/6J mice	Reduced DSS-induced inflammation Decreased IL-6 and TNF-α levels	[83]
Allicin	25 and 50 mg/kg	Colon samples from Sprague Dawley Rats	Decreased acrylamide-induced LPS levels Decreased levels of IL-1β, IL-18, TNF-α, and IL-6 Increased IL-10 levels Upregulated tight-junction proteins expression	[43]
Berberine	10 and 20 mg/kg	Colon samples from C3H/HeN mice	Reduced 2,4,6-trinitrobenzenesulfonic acid-induced colitis Decreased IL-1β, TNF-α, and IL-6 levels Increased IL-10 levels Inhibited TLR4, iNOS and COX-2	[90]
Caffeic acid	60 and 120 mg/kg	Intestinal samples from Sprague Dawley Rats	Reduced ketoprofen-induced NO levels and COX-2 expression	[79]
Kaempferol	50 mg/kg	Colon samples from C57BL/6J mice	Reduced DSS-induced colitis Reduced serum levels of IL-1β, IL-6, and TNF-α Increased IL-10 mRNA expression Decreased mRNA expression of IL-1β, IL-6, COX-2, iNOS, TLR4, NLRP3, MAPK1, and NF-kB Increased mRNA expression of ZO-1, occludin and claudin-1	[91]
Eriodictyol	20 and 50 mg/kg	Colon samples from Wistar rats	Reduced 2,4,6-trinitrobenzenesulfonic acid-induced colitis Increased IL-10 levels Decreased levels of IL-1β, IL-12, IL-2, TNF-α, and IL-6 Reduced TLR4 expression and NF-kB activation	[82]
Naringin	25–100 mg/kg	Colon samples from C57BL/6J mice	Reduced DSS-induced colitis Reduced IL-1β, TNF-α, and IL-6 levels Decreased NF-kB activation	[92]
Chlorogenic acid	25 and 50 µM	Porcine intestinal epithelial cells (IPEC-J2)	Reduction in LPS-induced TNF-α, IL-8, and IL-6 encoding genes expression and IL-8 and IL-6 levels Reduced COX-2 expression	[84]
Resveratrol	10–50 µM	Human colorectal adenocarcinoma cells (Caco-2)	Reduced LPS-induced COX-2 protein and mRNA expression Reduced PGE_2_ production Inhibited NF-kB pathway	[93]
3-Acetyl-11-keto-β-boswellic acid	27 ng/mL	Human colorectal adenocarcinoma cells (Caco-2)	Reduced TNF-α/IFN-γ-induced downregulation of tight-junction proteins (ZO-1 and occludin) Downregulated NF-kB expression Reduced paracellular permeability induced by inflammatory stimuli	[59]
Schisandrin A	10 µM	Human colorectal adenocarcinoma cells (HT-29)	Reduced deoxynivalenol-induced inflammation Decreased COX-2, NF-kB, and MAPK expression Reduced NO, IL-8, and PGE_2_ levels	[85]
Cyanidin-3-*O*-glucoside	0.05–0.2 µM	Human colorectal adenocarcinoma cells (Caco-2)/mouse macrophages (RAW 264.7) co-culture	Reduced LPS-induced TNF-α, IL-1β, IL-6, and IL-8 levels in the apical side of transwell model	[94]

Chlorogenic acid, in addition to the capacity to improve the redox status of porcine intestinal epithelial cells (IPEC-J2) stimulated with LPS (Table 2), was able to reduce the production of several cytokines (TNF-α, IL-8, and IL-6) as well as COX-2 expression in the same model, at 25 and 50 µM for a low exposure time (1 h) [84]. As a relevant note, the cell viability assay revealed that chlorogenic acid is not toxic at concentrations up to 50 µM for 1 h (same exposure time used for the assays described above); however, it presented toxicity for exposure times ≥ 4 h [84], which is a reasonable time period for the phenolic acid to be in contact with the intestinal tract, given that chlorogenic acid is found in many products such as coffee, tea, cocoa, and fruits [84,95].

EGCG has also been studied as a potential dietary agent to mitigate oxidative damage and inflammation at the intestinal level. Wu et al., 2021 [83] evaluated its protective effect in mice’s colon with DSS-induced colitis, both as a preventive measure and as treatment, and found that EGCG was effective in reducing TNF-α and IL-6 concentration in the colon epithelium, as well as IL-1β, IL-6, IL-8, and TNF-α in the plasma, either when administered before or after colitis induction [83]. In a similar pattern, SOD and GPx levels were increased in the colon, in both EGCG treatments, and in prophylactic EGCG administration, CAT was also elevated, and lipid peroxidation was decreased. Also, EGCG modulated the gut microbiota, particularly promoting an increase in short-chain fatty acids-producing bacteria [83]. These fatty acids are by-products of the fermentation of dietary fibre by several bacteria present in the intestinal tract that are being studied for their role in intestinal barrier homeostasis [96,97,98]. Some short-chain fatty acids were reported for their potential in the treatment of inflammatory bowel diseases and tumours, as well as to present antioxidant potential, to target NF-kB and MAPK signalling pathways, the endogenous enzymatic antioxidant systems, and cell proliferation/death signalling axis [96,97,98].

Phytochemicals also present the potential to protect against inflammation caused by foodborne mycotoxins. Schisandrin A (or deoxyschizandrin), a dibenzocyclooctadiene lignan found in the fruit of *Schisandra chinensis* (Turcz.) Baill is able to protect against the oxidative damage and inflammation induced by deoxynivalenol, a mycotoxin produced by fungi from the *Gibberella* genus [85]. In addition to the reduced ROS and MDA levels, with enhanced activity of endogenous antioxidant enzymes, Schisandrin A reduced nitric oxide, IL-8, and PGE_2_ levels, reduced COX-2 expression and reduced deoxynivalenol-induced activation of NF-kB and MAPK in HT-29 cells [85]. Thus, phytochemicals and other natural compounds have the ability to protect the gastric tract against several sources of oxidative stress and inflammation by targeting specific signalling pathways involved in these processes.

## 3. Main Molecular Targets in Antioxidant and Anti-Inflammatory Response

**Figure 2:**
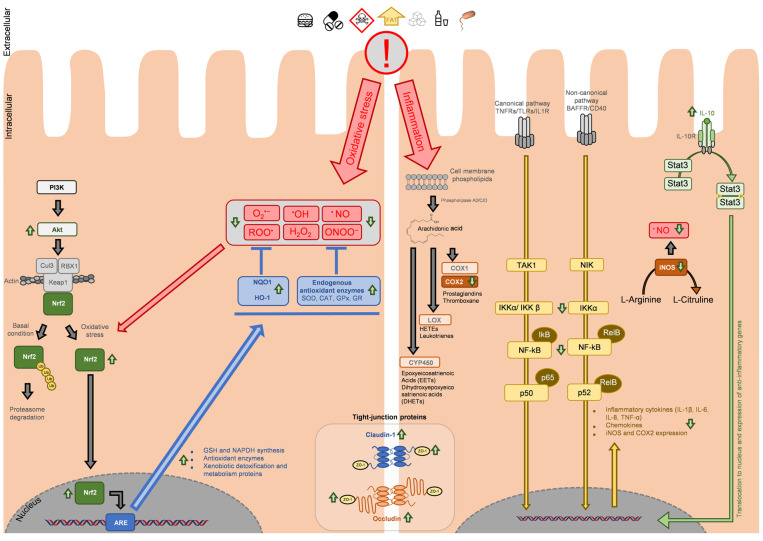
Simplified schematization of signalling pathways and the main targets involved in phytochemicals’ antioxidant and anti-inflammatory response at intestinal level as reported in the literature (Table 1, Table 2, Table 3 and Table 4). In response to the oxidative and inflammatory stimuli that arise from xenobiotics and pathogens, phytochemicals have been shown to upregulate/downregulate key proteins in both signalling processes. In oxidative stress, phytochemicals have been shown to upregulate PI3K/Akt/Nrf2 signalling axis, leading to Nrf2 translocation to the nucleus and enhanced transcription of ARE (antioxidant response element) genes, leading to increase in GSH production, expression of antioxidant enzymes, HO-1 and NQO1, and phase II detoxification proteins. This culminates in a reduction in reactive oxygen and nitrogen species. In inflammation, phytochemicals are shown to inhibit COX-2 and iNOS, resulting in a decreased production of prostaglandins and nitric oxide, respectively. NF-kB arises as the major target of bioactive molecules, whose inhibition suppresses the production of inflammatory cytokines. Additionally, phytochemicals have been shown to increase the expression of IL-10 expression, an anti-inflammatory cytokine, and to upregulate the expression of tight-junction proteins (claudin-1, occludin, and ZO-1). All abbreviations not defined here are defined above. Figure adapted from [25,99,100,101,102,103,104,105,106,107,108,109,110].

Within the cellular antioxidant activity, as observed in Table 1 and Table 2, Nrf2 is highlighted as the main target of phytochemicals in many studies. Figure 2 represents the main molecular targets of phytochemicals involved in the modulation of antioxidant and anti-inflammatory pathways at the intestinal barrier, summarized from the studies reviewed above.

Despite not being the only pathway described for Nrf2 activation, the literature analysis suggests the role of phytochemicals on PI3K/Akt-dependent Nrf2 activation [49,75,103,111,112]. Under homeostatic conditions, Nrf2 is linked to Keap1, which acts as a repressor and regulates Nrf2 activation. Keap1 mediates the formation of a complex with RBX1 (RING-box protein 1) and Cul3 (cullin 3), leading to Nrf2 ubiquitination and further proteasomal degradation. This mechanism ensures low levels of Nrf2 in basal conditions [99,100,101,102,104].

Under oxidative stress, ROS are able to module cysteine residues in Keap1, thus releasing Nrf2 and suppressing its ubiquitination. Once free, Nrf2 translocates and accumulates in the nucleus, where, upon dimerization with small Maf (musculoaponeurotic fibrosarcoma) proteins, it binds to DNA in ARE-regulated (antioxidant response element) genes, promoting GSH production, increasing the expression of several key proteins such as NQO1 (NAD(P)H quinone oxidoreductase 1), HO-1 (heme-oxygenase 1), GCLs, endogenous antioxidant enzymes (SOD, CAT, GPx, and GR), and phase II metabolization and detoxification enzymes, as well as stress response proteins [100,112,113]. Various phytochemicals have been reported as Nrf2 activators, as is the case of the polyphenols curcumin, EGCG, or genistein [100], found in the turmeric rhizome (*Curcuma longa*), green tea, or legumes (e.g., lupin, fava bean, and soybean), respectively [114,115].

Regarding the inflammatory cascade, an analysis of Table 3 and Table 4 allows for the identification of the major pathway that is a target of natural anti-inflammatory agents. This is the inhibition of NF-kB pathway activation, which is an upstream enhancer of prostaglandins, cytokines and other inflammatory mediators, which are also targeted by phytochemicals [105,106]. The NF-kB pathway is interconnected with a variety of pathways and can play many roles in homeostatic regulation, such as cell proliferation, maturation and differentiation, but also in colorectal cancer tumourigenesis, and particularly relevant in this review, in inflammation and immune response [105,106]. NF-kB pathway activation in inflammation is usually described as dependent on ligand binding to cell membrane receptors. Depending on the ligand/stimuli and the receptor, NF-kB activation may follow the canonical or non-canonical pathways. TLRs, TNFRs (tumour necrosis factor receptors), or IL-1R (interleukin-1 receptor) trigger the canonical pathway via TAK1 (mitogen-activated protein kinase kinase kinase 7 (MAP3K7)), while BAFFR (B-cell activating factor receptor) or CD40 (cluster of differentiation 40) trigger the noncanonical pathway via NIK (NF-kB-inducing kinase; or mitogen-activated protein kinase kinase kinase 14, MAP3K14) [25,105,106].

Regardless the pathway, the modulation of IκB kinase complex (inhibitor of nuclear factor kappa-B kinase subunit alpha, beta and gamma (IKKα, IKKβ and IKKγ)) is necessary, and the inflammatory cascade progression leads to NF-kB translocation to the nucleus, where it accumulates and promotes the expression of proinflammatory cytokines, chemokines, iNOS and COX-2 [25,105,106].

COX-2 is then part of the arachidonic acid pathway, a polyunsaturated fatty acid present in the cell membrane’s phospholipids. Under inflammatory stimuli, phospholipids are released from the cell membrane and hydrolyzed by phospholipases (mainly phospholipase A2 (PLA_2_) or PLC), releasing arachidonic acid. This molecule is then used as a precursor for the synthesis of epoxyeicosatrienoic acids (EETs) and dihydroxyepoxyeicosatrienoic acids (DHETs) by cytochromes P450 (CYP450), of hydroxyeicosatetraenoic acids (HETEs) and leukotrienes by lipoxygenases (LOX), or prostaglandins and thromboxane by COX-1 and COX-2 [107,108]. COX-2, as seen above, is a major target of natural compounds (Table 3 and Table 4).

Among cytokines, the pro-inflammatory cytokines IL-1β, IL-6, IL-8, and TNF-α assume a high preponderance in inflammation. IL-8 is of particular interest, given its association with IBDs, but also for its role in colorectal cancer [116,117,118,119,120].

Nevertheless, the upregulation of anti-inflammatory cytokine IL-10 arises as a determinant factor in many studies. This cytokine is produced by immune and epithelial cells; in fact, the majority of cells that express IL-10 also express its receptor (IL-10R), implying that an autocrine regulation is possible [18,19,121]. Within the intestinal mucosa, immune cells (lymphocytes and macrophages) are the main source of IL-10, but its production by epithelial cells is also described [18,19,121]. Regarding IL-10R, its expression and location differ slightly. The activation of this receptor is initiated by IL-10 binding, leading to STAT3 (signal transducer and activator of transcription 3) dimerization and translocation to the nucleus (Figure 2) [110]. Its expression in human intestinal epithelial cells is reported, although studies in human colon cell lines showed that the expression of the receptor mRNA is not present in all cell line models [18]. This is relevant to mention, as it highlights the necessity to consider the influence of the immune system in the inflammatory process at the intestinal level when designing in vitro studies with a single-cell model and the necessity to not generalize the effect singly on epithelial cells when analyzing intestinal samples of in vivo models.

Regarding IL-10R location, this differs according to the source of the stimuli, as IL-10R expression was observed in the apical membrane of human colon epithelial cells, presumed to bind IL-10 produced by these cells [18].

Considering the mucosal anatomy, the epithelial cells assume a polarized alignment, where the apical membrane faces the lumen, while the basolateral membrane faces the *lamina propria*, which contains the immune cells that produce the majority of IL-10, and thus, assuming the integrity of intestinal barrier, IL-10 should bind to receptors in the basolateral membrane [18]. Nevertheless, to the question if there is or is not a different response caused by IL-10 binding in apical or basolateral membranes, there is no answer yet, which should be addressed in future studies. Regarding homeostasis, IL-10 upregulates tight-junction proteins, inhibits the production of pro-inflammatory cytokines, prevents epithelial cell apoptosis, and its deregulation is significant in the development of IBD [18,19,121,122,123,124].

It must also be highlighted the high number of studies reviewed above regarding natural products that present both antioxidant and anti-inflammatory activities, which is not surprising since there is a significant crosstalk between the pathways illustrated in Figure 2. This is related to Nrf2’s role in inflammation, where it can reduce LPS-induced ROS and cytokine production [100,125,126,127]. It was shown that colon samples from DSS-induced colitis mice model with Nrf2 knockout presented higher severity of the pathology when compared to the wild-type mice [100]. This is likely to be, at least in part, correlated with the indirect interaction between Nrf2 and NF-kB that presents itself under different molecular mechanisms. Upon upstream signalling, both Nfr2 and NF-kB translocate and accumulate in the nucleus when they require binding to CREB-(cAMP response element-binding protein)-binding protein (CBP), which creates a competition of both factors for CBP, determined by the accumulation of each one [100,125,126,127]. Different crosstalk is related to reciprocal inhibition of each pathway end-products over the other pathway; more precisely, proteins whose expression is mediated by ARE genes (such as HO-1 or SOD) are able to inhibit the progression of NF-kB transcription. Additionally, anti-inflammatory agents that downregulate NF-kB may also present Nrf2 upregulation, although the inverse action is also described. Lastly, NF-kB is also able to inhibit Nrf2 via HDACs (histone deacetylases), preventing the expression of ARE genes [100,125,126,127]. Nrf2’s pivotal role also extends to the regulation of tight-junction proteins, being able to upregulate the expression of ZO-1, occludin, and claudin-1 [127]. The study of the Nrf2 pathway and the crosstalk with other signalling pathways that regulate intestinal barrier homeostasis and with the inflammatory cascade assumes a major role, with potential pharmaceutical applications for novel activators of this protein.

## 4. The Role of Macrophages in Oxidative Stress and Inflammation Management in the Intestinal Barrier

A different topic is related to the relevance of the immune system in intestinal tract inflammation, as the impact of natural products and phytochemicals in immune cells cannot be overlooked. In fact, regarding the study of the anti-inflammatory activity of dietary products, immune cells such as macrophages represent a large portion of studies performed, especially using the cell models RAW 264.7 or J774A.1 (mouse macrophages) and THP-1 (human monocytes). Among the dietary products commonly consumed, whose anti-inflammatory potential was reported for these cell lines, not only can numerous extracts obtained from aromatic and medicinal plants be found (such as *Mentha* spp. [128,129,130], *Lavandula* spp. [131,132,133], *Coriandrum* spp. [134], *Salvia* spp. [135,136,137], *Thymus* spp. [138,139,140,141], or *Origanum* spp. [142,143,144]) but also from vegetables (e.g., green lettuce (*Lactuca sativa* L.) [145], cabbage (*Brassica* spp.) [146,147]), and from other foodstuff, such as olive oil [148], cinnamon [149], edible mushrooms [150], fruits [151,152,153], fruit juices [154,155], or coffee [156,157,158]. From these studies, the ones addressing signalling pathways involved in the anti-inflammatory response highlight the downregulation of the same main molecular targets observed above for intestinal epithelium, i.e., NF-kB, iNOS, COX2, IL-1β, and IL-6, highlighting the specificity of natural molecules for these cellular biomarkers of inflammation.

This is also observed for individual phytochemicals tested in RAW 264.7, J774A.1, or THP-1 cell lines that are commonly found in the products mentioned above, some of which were also effective in intestinal inflammation models, as seen in Table 3 and Table 4. Among a high number of molecules fitting this description, we can find phenolic acids, flavonoids and terpenoids, for example. To name some of the most common ones, EGCG [159,160], quercetin and its derivative rutin [161,162,163,164], luteolin and its derivatives luteolin-7-*O*-glucoside and luteolin-7-*O*-glucuronide [161,165,166], kaempferol [161], apigenin [161], eriodictyol [167], rosmarinic acid [140,164], caffeic acid [136,168], oleanolic acid [169], ursolic acid [169,170], or carvacrol [171] are examples.

The relevance of these studies in the present review is related to the major role played by macrophages in both intestinal epithelium homeostasis and inflammation. Due to its role as a barrier separating the xenobiotics and pathogens in the intestinal lumen from the systemic circulation, within the intestinal system is contained the biggest single section of the immune system and the largest population of macrophages, a fact arising from the vast superficial area displayed by the intestinal tract when compared to other organs in the human body with significant presence of the immune system [172]. Regarding their distribution, the largest portion of the intestinal barrier macrophages are present in the *lamina propria*, being a large portion of the leukocytes present in this layer of the intestinal mucosa [172]. Comparing intestinal tract sections, the colon’s *lamina propria* presents a higher macrophage count when compared to the duodenum, jejunum, and ileum. Nevertheless, this type of mononuclear phagocytes can also be found in smooth muscle cells, despite in smaller numbers and with function related to intestinal motility [172]. Within the *lamina propria*, different expressions of surface markers (for example, CX3CR1 (CX3C motif chemokine receptor 1), MHCII (major histocompatibility complex II), or Ly6C (Ly6c1 lymphocyte antigen 6 family member C1) can be used to differentiate macrophages and its function in rodents’ intestinal tract [172,173,174,175]. In humans, some of these markers can also be used, but the classification is not precise, as macrophages often adapt and express markers outside the classical and less flexible classification [172,173,174,175].

In homeostasis, gut macrophages regulate the proliferation of epithelial progenitor cells in the crypts, present constitutive expression of PGE2, as well as IL-10, which regulate other immune cells, control ROS and TNF-α production by neutrophils, provide clearance of apoptotic and senescent cells, and participate in tissue remodelling [172,173,175]. It is relevant to mention that despite its major role as an inflammatory cytokine, constitutive TNF-α action can extend to enterocyte growth, tissue remodelling, and barrier permeability regulation, among other functions [172]. It was also reported that when selectively depleted, the absence of macrophages increases the effects of induced acute colitis in mice [176]. Interestingly, intestinal tract macrophage populations can differ from their homologous in other body parts in response to bacteria, LPS, or other bacterial by-products. These cells present increased phagocytosis but are less sensible to TLR and NOD receptors activation. There is no upregulation of pro-inflammatory cytokine production or nitric oxide release, and they prevent scaling of the inflammatory cascade triggered by commensal bacteria [172,173,174].

In inflammation, the macrophages are responsible for the phagocytosis of bacteria and other exogenous materials that evade the most external layer of the intestinal mucosa [173]. There is an accumulation of pro-inflammatory monocytes, which differentiate into macrophages with higher sensitivity to bacteria and their metabolites and enhanced cytokines production [173,177]. However, the macrophages responsible for homeostasis and anti-inflammatory stimuli maintain their role during the inflammatory event, highlighting the different populations present at the intestinal level as a result of different differentiation and observed by the different expression of the surface markers described above [173,177]. Even more, macrophage population deregulation is linked to the decreased tolerance to food antigens and gut microbiome [173]. In IBD, recent studies point to alteration in the role of macrophages in inflammation clearance, where deregulation in monocyte–macrophage differentiation leads to enhanced cytokine production and less effective bacterial clearance, thus prolonging the inflammatory event and leading to the symptoms observed in these pathologies [173,177].

While both rodents and humans present higher macrophage count in the colon when compared to the small intestine, it was observed that in rodents, the increasing count of macrophages is observed in the full extension of the intestinal tract (from proximal to distal sections), while human colon presents identical presence of these cells in its extension [172]. However, it was reported that macrophage populations from human and rodent intestinal tracts present various similarities [173], which supports the use of mouse in vitro cell models, up to a certain degree, to study highly conserved pathways in the pro- and anti-inflammatory signalling cascades, especially in samples such as natural compounds that are mostly understudied.

Therefore, despite the focus of this review is the inhibition of the inflammatory cascade in enterocytes, the role of the immune system cannot be overlooked in in vivo assays. In these latter, mainly using mice/rats, the analysis of biomarkers could be extended to intestinal epithelial cells and the immune cells in *lamina propria*, rather than just ileum, jejunum, or colon epithelial sections, to provide a better understanding of the source of inflammatory stimuli and target of the natural compounds’ inhibitory activity.

### 4.1. Inflammatory Bowel Diseases (IBD), Current Available Treatments, and the Use of Phytochemicals in Preventing and Mitigating the Symptoms

Considering the clinical treatment of intestinal inflammation, several options are available due to the high incidence of Crohn’s disease and ulcerative colitis, although the available drugs often only control the symptoms. Therapeutic approaches are mainly based on corticosteroids, specific immunosuppressants, biologic agents or aminosalicylates, being the last the most common drugs prescribed in IBD treatment [178]. Among biological agents can be found options such as infliximab, a monoclonal anti-TNF-α antibody (a drug designed to reduce TNF-α binding to its receptor), or vedolizumab, a recombinant humanized IgG1 monoclonal antibody, which reduces T-cells recruitment. However, a large percentage of individuals with inflammatory bowel diseases develop a resistance to anti-TNF therapy, and the mechanism of action of these biological agents is still partly unclear [179]. Additionally, this therapy presents serious side effects, such as an increased risk of infections [180]. The main therapeutic approach still resides in the use of aminosalicylates, such as 5-aminosalicylic acid (known as mesalazine), a COX inhibitor [181]. The main limitations of these drugs are the need to trial between the different 5-ASAs for each patient, which often limits the effect of the treatment implying high costs and side effects that include kidney, heart, lung, and pancreas diseases [182]. Thus, other options are needed.

Aiming for IBD mitigation, various studies proposed the use of natural compounds targeting the intestinal macrophages. This is the case of fraxinellone, a lactone identified in various plant species, which was observed to target the NF-kB pathway and inflammasome activation in macrophages from DSS-induced colitis mice model [183]. The therapeutic effect was related to the inhibitory activity over macrophage infiltration, resulting in a reduction in pro-inflammatory cytokines (IL-1β, IL-6, and TNF-α) in the colon, as well as inhibition of iNOS and COX-2. When studying the replication of the result in in vitro assays using THP-1 cell line stimulated with LPS, the authors observed a decrease in NO, IL-1β, and IL-18 levels, as well as iNOS inhibition [183].

A different study using a procyanidin (not specified) reported the inhibition of the NF-kB pathway in a colitis model, preventing the activation of M1-type macrophages from a DSS-induced colitis mice model, with decreased production of cytokines and iNOS expression [184]. In similar manner, the triterpenoid toosendanin (extracted from *Melia toosendan* Sieb et Zucc) was found to also reduce M1-type macrophages activation, mainly via inflammasome inhibition (by targeting NLRP3), which resulted in diminished production of inflammatory cytokines, but also upregulating Nfr-2 and HO-1 expression in both the macrophages and colon tissue of C57BL/6 mice stimulated with DSS-induced colitis [185]. Another study using caffeic acid, also in macrophages from C57BL/6 mice stimulated with DSS, showed that the phenolic acid inhibited macrophage infiltration in the intestinal mucosa [186].

Han et al., 2023 [187] studied the effect of diet enrichment with phenolic acids in the mitigation of intestinal inflammation. The authors observed that the anti-inflammatory effect observed was dependent on the phenolic used, where chlorogenic acid inhibited NLRP3 activation and pyruvate kinase M2-dependent glycolysis in macrophages. Ferulic acid acted on neutrophils, while caffeic acid and ellagic acid modulated the gut microbiome, all converging to a reduction in colitis. Ellagic acid, in particular, is metabolized into urolithin A by gut bacteria. This metabolite also showed potential to mitigate colitis and promote barrier homeostasis, unlike the other ellagic acid metabolite urolithin B, highlighting the role of gastrointestinal digestion in the bioactivities observed [187].

Recent studies are refining the methodologies available to better understand the crosstalk between intestinal epithelial cells and macrophages via cell-based in vitro assays, using trans-well inserts and companion plates, where epithelial cells (usually Caco-2 cells) are seeded in the insert and then differentiate and polarize to present an apical membrane in the upper chamber and basolateral membrane facing to the lower chamber. At the bottom of the plate are seeded macrophages (usually RAW 264.7 cells). This model intends to simulate the epithelial monolayer and macrophages from *lamina propria*, as only the enterocytes contact with the apical compartment (simulating intestinal lumen), the products of intestinal absorption and metabolization proceed to the basolateral compartment, which is also in contact with the macrophages, allowing the cytokines produced from these later to be in contact with the enterocytes. A study reports the effect of acai berry extract on this type of model, where inflammation was induced by LPS, added to the basolateral compartment, which, as expected, increased cytokine production [188]. Upon treatment with the extract in the apical chamber, tight-junction proteins were upregulated in Caco-2 cells (ZO-1, JAM-1 (junctional adhesion molecule 1) and claudins). The analysis of the supernatant of Caco-2 cells revealed a decrease in IL-6, IL-8, and TNF-α expression, and cell protein analysis showed a decrease in p65, p38, JNK, and ERK phosphorylation [188]. A similar study reported the potential of polysaccharides from *Ganoderma atrum* [189], where also adding the sample to the apical chamber and LPS to the basolateral chamber, it was observed that IL-6, IL-1β, TNF-α, and ROS levels were decreased, COX-2 expression was reduced, as well as the phosphorylation of p38, JNK and ERK, while upregulating Nrf2 pathway [189].

Using only human cell lines, the anti-inflammatory activity of a coffee leaf extract was evaluated in a co-culture of Caco-2 cells and U937 cells (human monocytes) [190]. U937 were differentiated into macrophages using phorbol 12-myristate-13-acetate, pre-incubated with the extract, and then the co-culture was stimulated with LPS. While no changes were observed in the TNF-α level in the apical chamber, a slight decrease was observed in the basolateral chamber. IL-1β was not detected in the apical chamber (with or without LPS exposure), but its levels were significantly reduced in the basolateral chamber [190]. Interestingly, IL-8 levels were reduced in both the apical and basolateral chambers [190]. These studies provide advantages when compared to studies using RAW 264.7, related to IL-8 expression, since rodents do not present IL-8 encoding gene [191] and also present differences in IL-8 receptors expression [192], and therefore are not able to produce this cytokine, limiting its study.

### 4.2. Limitations in Using In Vitro vs. In Vivo Models

Highly relevant when considering in vitro vs. in vivo studies is the fact that cell models-based assays often do not consider the effect of gastrointestinal digestion, while in in vivo assays where the natural product is given orally, this factor is always present, adding additional complexity to the assay. When compared to tissues that are dependent on intestinal absorption, bioaccumulation, and systemic circulation to be in contact with the phytochemicals ingested, the intestinal tract benefits from not being dependent on these parameters, being exposed to the full content in the diet. However, this does not exclude the effect of the digestive process, which can modulate the composition and quantity of the phytochemicals that, in fact, make contact with the intestinal barrier and thus affect its bioactivities. Lipid nanoparticles as a solution to overcome the low bioavailability and further intestinal absorption of extracts and individual phytochemicals [193], which can be helpful for the development of pharmaceutical products.

Several studies have addressed this topic, mainly aiming to understand if the antioxidant potential of various natural products commonly presented in the diet is affected by the digestive process. Among those, Martínez-Las Heras et al., 2017 [194] reported that the antioxidant potential (evaluated via colorimetric assay for synthetic radical scavenging; DPPH) of persimmon leaves’ aqueous extract decreased during the buccal and gastric phases of the digestive process. Despite the solubility of polyphenols in the simulated intestinal fluid being enhanced by the pH = 7 and the presence of pancreatin and bile salts, the overall antioxidant activity was decreased when compared to the undigested extract. The study also highlights that, although the undigested extract of persimmon leaves presents higher antioxidant potential than the persimmon fruit, after digestion, the fruit is able to provide higher polyphenol yield, and both products present similar antioxidant potential in the intestine phase. In addition, the presence of digestive enzymes in the simulation greatly favours the polyphenols’ bioavailability [194].

A similar study was conducted using an ethanolic extract of *Prunus spinosa* L. (blackthorn) branches [195], where the authors reported different outcomes, stating that the buccal and gastric phases had no significant effect on the polyphenols present in the extract, whilst in the intestinal phase, a significant metabolization of the original compounds was observed. The antioxidant potential decreased in each phase of the in vitro simulated digestion process [195]. Regarding the bioavailability of individual components from complex matrices (such as extracts), D’Antuono et al., 2015 [196] studied the bio-accessibility and bioavailability of phytochemicals present in aqueous and hydroethanolic extracts of artichoke (*Cynara cardunculus* L.). The authors selected aqueous extraction due to its similarity to the extraction process in the digestive process when compared to organic solvents. Within the main components of the extract, the study reveals that gastric and intestinal phases of the digestive process reduce the content of chlorogenic acid to 70%, 3,5-*O*-dicaffeoylquinic acid to 41.3% and 1,5-*O*-dicaffeoylquinic acid to 50.3%, comparing to the starting content [196]. In addition, the maximum accumulation of flavonoids was observed after 30 min of exposure, using Caco-2 cells, and with an efficiency as low as 0.16%, and therefore although it was observed antioxidant activity for these extracts (evaluated using LDL oxidation assay as an in vitro approach to lipid peroxidation), it is concluded that an extrapolation cannot be performed due to the effect of the digestive process in the components that reach the intestinal tract [196].

## 5. The Need to Find Correlations between Experimental Data and Clinical Effect and Future Directions

Aiming to correlate the effects observed in the various reported in vitro and in vivo (using animal models) works, several clinal trials are being performed, but most of them do not yet have published results. For example, the clinical trial “Curcumin for Prevention of Relapse in Patients With Ulcerative Colitis” (ClinicalTrials.gov ID NCT03122613), where, due to the anti-inflammatory action of curcumin, and associated with its anti-oxidant and anti-tumour properties [88,89], is currently being studied as an alternative treatment to ulcerative colitis. Another example is the ongoing clinical trial concerning the effect of the Mediterranean diet on IBD biomarkers by assessing serum and stools specific biomarkers in individuals subjected to 42 days of a specific diet (ClinicalTrials.gov ID NCT05973500; “Effect of Mediterranean Diet in Inflammatory Bowel Disease”). This study has no conclusions yet.

As future directions, these studies highlight the need to refine in vitro and in vivo studies to better depict the antioxidant activity of foodstuff tested in biological systems, as the colorimetric assays based on chemical scavenging of free radicals are often poor predictors of the potential involved in modulating intestinal cells response to oxidative stress. The same principle can be applied to inflammation studies, as the digestive process and gut microbiome may alter the composition/structure of the bioactive molecules under study, limiting the understanding of the observed effect in in vivo assays or providing a result in in vitro assays that will not be reproducible in the whole organism, and ultimately cannot be correlated to humans’ physiology. Thus, the resources for clinical trials will permit the assessment of the physiological effects of phytochemicals in reducing IBD biomarkers. However, the implementation of balanced diets (e.g., based on Mediterranean diets) which supply the essential nutrients (micro and macronutrients) and additionally supply equilibrated quantities of a variety of phytochemicals will help improve gut microbiota balance as well as intestinal tract barrier integrity and functionality.

## 6. Conclusions

In the present review, we summarize recent findings regarding the antioxidant and anti-inflammatory potential of natural products and their components in the intestinal barrier. Due to the high exposure to a growing number of pathogens and xenobiotics through food, it is necessary to find new functional foods and nutraceuticals that may help to maintain intestinal barrier homeostasis. Nfr2 and NF-kB assume the role of major targets in these bioactivities, with their upstream and downstream signalling pathways comprising a wide range of other proteins targeted by phytochemicals. Despite the growing interest, there is still a large gap of information separating scientific knowledge from therapeutic application, a problem that may be solved with the refinement of in vitro and in vivo experimental models that can correctly predict the potential of these products, especially in the mitigation of inflammatory bowel diseases, where the complexity of signalling pathways between epithelial and immune cells is a limitation. On the other hand, the implementation of balanced diets (e.g., Mediterranean diet) containing a variety of food products, emphasizing those of plant origin, will provide a variety of natural molecules (e.g., phytochemicals) that have a positive effect on the prevention and mitigation of several diseases of the intestinal tract and others.

## Figures and Tables

**Table 1:** Antioxidant activity of various natural products, administered as extracts, evaluated in vitro and in vivo experimental models of the intestinal tract.

Plant/Extract	Concentration	Experimental Model	Observations	Ref.
Grape pomace aqueous extract	5 g extract/100 g diet	Duodenum lysate from swine	Reduced lipid peroxidation Increased antioxidant potential Increased CAT and GPx activities	[55]
		Colon lysate from swine	Reduced lipid peroxidation Increased antioxidant potential Increased SOD activity	
Grape seed proanthocyanidin extract	50 mg/kg	Colon samples from male C57BL/6J mice	Reduced dextran sulphate sodium-induced oxidative stress Decreased malondialdehyde production Normalized SOD activity Prevented GSH depletion	[56]
*Origanum vulgare* L. essential oil	5 and 20 mg/kg	Jejunum samples from Wistar rats	Reduced diquat-induced oxidative stress Decreased ROS and TBARS levels Normalized SOD and GPx activities	[47]
Fu brick tea (fermented tea; *Camellia sinensis* L.)	100 mg/kg	Colon samples from Sprague Dawley rats	Reduced oxidative stress induced by high-fat diet Decreased lipid peroxidation Increased SOD and CAT levels Reversed the downregulation of ZO-1, occludin, and claudin-1	[51]
Garlic oil	20 and 40 mg/kg	Colon samples from Sprague Dawley rats	Reduced alcohol-induced oxidative stress and lipid peroxidation Increased SOD and GPx levels Upregulated ZO-1 and Claudin-1 expression	[57]
*Astragalus membranaceus* dried root extract (Axtragyl^®^)	50 and 100 µg/mL	Rat small intestine epithelial cells (IEC-6 cells)	Reduced H_2_O_2_-induced ROS increase Activation of Nrf2 to nuclei Increased expression of HO-1 and NQO1	[58]
*Origanum vulgare* L. essential oil	10 µg/mL	Porcine small intestinal epithelial cells (IPEC-J2)	Reduced H_2_O_2_-induced intracellular and extracellular ROS increase Reduced lipid peroxidation Increased GSH and upregulated SOD, CAT, GCL, and Nrf2 expression	[48]
Cranberry (*Vaccinium macrocarpon* Aiton) extract *	200 mg/kg	Jejunum samples from male C57BL/6J mice	Reduced oxidative stress induced by high-fat/high-sucrose diet Increased SOD2 levels	[50]
Olive oil phenolic extract	25 µg/mL	Human colorectal adenocarcinoma cells (Caco-2)	Decreased oxysterols-induced ROS increase Prevented GSH depletion	[46]
*Boswellia serrata* resin (hydroethanolic extract)	1 µg/mL	Human colorectal adenocarcinoma cells (Caco-2)	Reduced H_2_O_2_-induced ROS increase and NF-kB expression Prevented downregulation of tight-junction proteins (ZO-1 and occludin)	[59]
Cocoa extract	10 µg/mL	Human colorectal adenocarcinoma cells (Caco-2)	Reduced acrylamide-induced oxidative stress and cell death Prevented GSH depletion Increased GST and GCL levels	[42]

Notes: * standardized extract obtained from Nutra Canada (Quebec, QC, Canada); Abbreviations (not defined previously): NQO1, NAD(P)H quinone dehydrogenase 1; TBARS, thiobarbituric acid reactive substances.

**Table 3:** Anti-inflammatory potential of natural products, administered as extracts at the intestinal level.

Plant/Extract	Concentration	Experimental Model	Observations	Ref.
Fu brick tea (fermented tea; *Camellia sinensis* L.)	100 mg/kg	Colon samples from Sprague Dawley rats	Decreased LPS in serum induced by high-fat diet Reduced IL-6, TNF-α, and MCP-1 expression Increased IL-10 levels	[51]
Grape seed proanthocyanidin extract	50 mg/kg	Colon samples from C57BL/6J mice	Reduced dextran sulphate sodium-induced inflammation Decreased TNF-α and IL-1β levels and respective mRNA expression Restored IL-10 level and increased its mRNA expression Reduced mRNA expression of NLRP3, ASC, and caspase-1	[56]
*Origanum vulgare* L. essential oil	5 and 20 mg/kg	Jejunum samples from Wistar rats	Reduced diquat-induced TNF-α, IL-1β and IL-6 mRNA expression	[47]
Cranberry (*Vaccinium macrocarpon* Aiton) extract *	200 mg/kg	Jejunum samples from C57BL/6J mice	Reduced inflammation induced by high-fat/high-sucrose diet Reduced COX-2 and NF-kB expression	[50]
Polyphenol-rich extract of Zhenjiang aromatic vinegar		Colon samples from ICR mice	Reduced alcohol-induced inflammation Increased IL-10 and IL-22 levels Reduced TNF-α, IL-6, IL-1β, and LPS levels	[71]
*Astragalus membranaceus* Bunge. dried root hydroalcoholic extract (Axtragyl^®^)	50 and 100 µg/mL	Rat small intestine epithelial cells (IEC-6 cells)	Reduced IFN-γ/LPS-induced TNF-α release Inhibited nitrotyrosine formation Reduced iNOS and COX-2 expression Decreased NF-kB activation	[58]
*Punica granatum* L. fruit peel aqueous extract	50 µM GAE	Human colorectal adenocarcinoma cells (Caco-2)	Decreased ERK 1/2 activation Decreased NO release, IL-1β-induced NF-kB activation and IL-8 and PGE_2_ secretion	[87]
*Saccharum officinarum* L. stem aqueous extract	50 µM GAE	Human colorectal adenocarcinoma cells (Caco-2)	Decreased IL-1β-induced NF-kB activation, IL-8 secretion and PGE_2_ secretion	[87]
*Quercus robur* L. duramen aqueous extract	50 µM GAE	Human colorectal adenocarcinoma cells (Caco-2)	Decreased NO release, IL-1β-induced NF-kB activation and IL-8 secretion	[87]
*Vitis vinifera* L. seeds extract *	50 µM GAE	Human colorectal adenocarcinoma cells (Caco-2)	Decreased IL-1β-induced IL-8 secretion	[87]
*Theobroma cacao* L. extract *	50 µM GAE	Human colorectal adenocarcinoma cells (Caco-2)	Decreased NO release and IL-1β-induced PGE_2_ secretion	[87]
Olive oil phenolic extract	25 µg/mL	Human colorectal adenocarcinoma cells (Caco-2)	Decreased oxysterols-induced NO, IL-8 and IL-6 increase Reduced JNK and IkB phosphorylation Decreased iNOS expression	[46]
*Boswellia serrata* resin (hydroethanolic extract)	1 µg/mL	Human colorectal adenocarcinoma cells (Caco-2)	Reduced TNF-α/IFN-γ-induced downregulation of tight-junction proteins (ZO-1 and occluding) Downregulated NF-kB expression Reduced paracellular permeability induced by inflammatory stimuli	[59]

Abbreviations (not defined above): GAE, gallic acid equivalents; MCP-1, monocyte chemoattractant protein-1). Notes: * standardized extract not specified.

## Data Availability

Not applicable.
